# Pigmented basal cell carcinoma diagnosed in a dental setup: Report of a case with review of the literature

**DOI:** 10.1002/ccr3.7136

**Published:** 2023-03-27

**Authors:** Snehashish Ghosh, Safal Dhungel, Indu Bharkavi, Bhawana Subedi Sapkota, Prabesh Banstola

**Affiliations:** ^1^ Department of Oral Pathology College of Medical Sciences Bharatpur Nepal; ^2^ Department of Oral and Maxillofacial Surgery College of Medical Sciences Bharatpur Nepal; ^3^ Sathyabama University Dental College and Hospital Chennai India; ^4^ Department of Oral Medicine and Radiology College of Medical Sciences Bharatpur Nepal; ^5^ College of Medical Sciences Bharatpur Nepal

**Keywords:** basal cell carcinoma, pigmented, rare

## Abstract

Pigmented basal cell carcinoma is a rare variant of basal cell carcinoma, with only a limited number of reported cases. Because of its similar clinical presentation, it is often over‐diagnosed as malignant melanoma. Along with case presentation, the clinical, microscopic features, and differential diagnosis are discussed in this case report.

## Graphical abstract

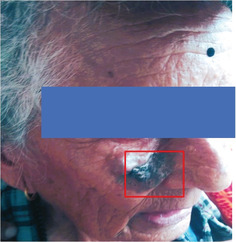

## 1 INTRODUCTION

Pigmented Basal cell carcinoma (BCC) is a rare variant of BCC, with only a limited number of reported cases. Because of its similar clinical presentation, it is often over‐diagnosed as MM. Along with case presentation, the clinical, microscopic features, and differential diagnosis are discussed in this case report.

Non‐melanoma skin cancers (NMSC) mainly consist of squamous cell carcinoma and basal cell carcinoma. They occur mostly in whites, but a fraction of cases also occur in dark‐skinned individuals.^1^

Basal cell carcinoma is the cutaneous neoplasm, frequently occurring in the sun‐exposed area of the head and neck, followed by the thigh and trunk.^2^, ^3^ It shows sluggish growth and tends to be locally invasive, burrowing into deeper structures.^2^, ^4^ Metastasis in BCC is rare, accounting for only 0.0028%–0.005% of the cases.^3^, ^5^ BCC has a multifaceted presentation that includes nodular, morpheaform, superficially spreading, cystic, and pigmented forms.^5^

Pigmented lesions, especially on the face are common, some present with classic pathognomonic features, and some appear to be deceptive.^6^ Pigmented basal cell carcinoma (PBCC) is one of the sparse variants of BCC, with only a small number of cases reported in the literature.^2^ It presents as an irregular pigmented patch, or plaque, where the color may vary from brown to black, making its diagnosis deceptive and commonly misdiagnosed as malignant melanoma (MM). However, its clinical course is nonviolent, the prognosis is much better than MM.^2^, ^7^

In this case report, we present a unique case of pigmented BCC, occurring in a 76‐year‐old South‐East Asian female, on the medial aspect of the face.

## 2 CASE REPORT

A 76‐year‐old female patient was accompanied by her son to the oral and maxillofacial surgery department of the College of Medical Sciences, Bharatpur Nepal. The patient presented with a chief complaint of toothache. While recording the history of the patient, extraorally, a hyperpigmented lesion was observed on the face. Following the redressal of her chief complaint, the patient was made aware of the extraoral lesion and was convinced to present a detailed history about it, but the patient and the attendees denied any treatment.

**FIGURE 1:**
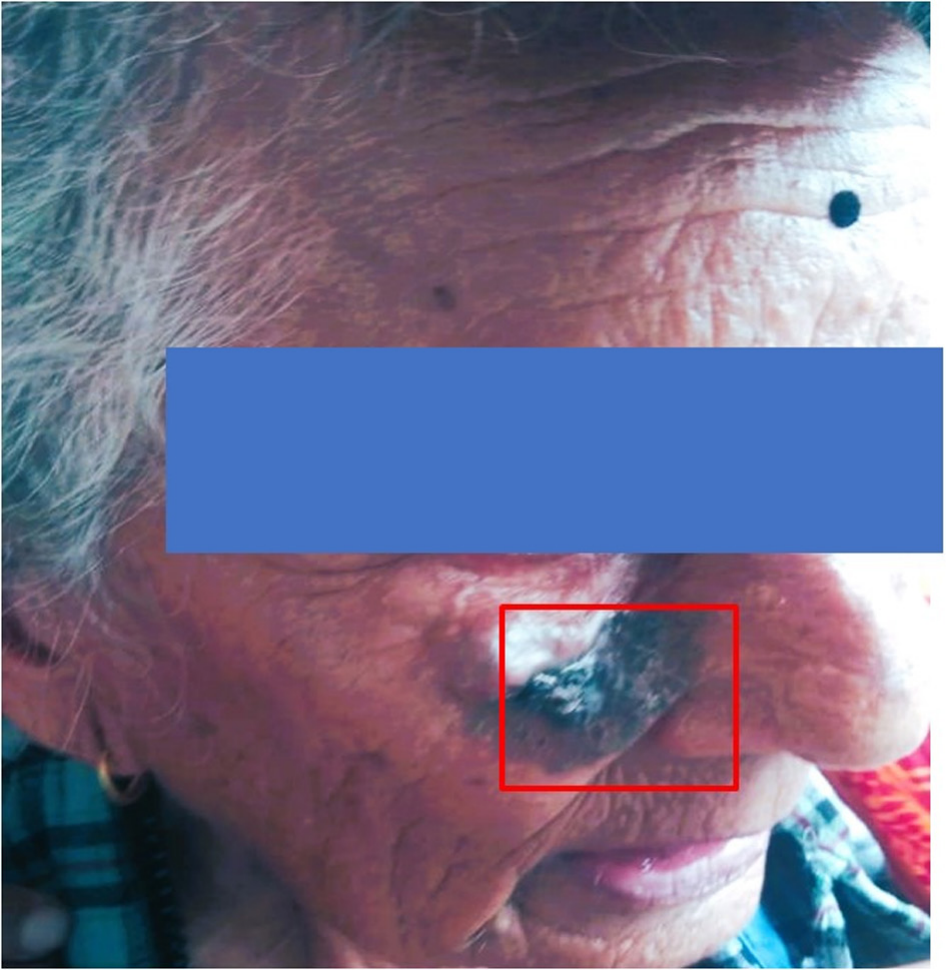
An irregular hyperpigmented patch on the medial aspect of the face, accompanied by a small, irregular nodule.

The patient revealed that it initially began 3 years back as a pea‐sized irregular dark‐colored patch and gradually increased to the present size. The lesion was neither painful nor associated with any kind of discharge. On clinical examination, an irregular hyperpigmented patch measuring about (2.5 × 1.5) cm was noted on the medial aspect of the face, lateral to the nose accompanied by a small irregular nodule. (Figure 1) The lesion was non‐tender, not fixed into the underlying deeper structures. Based on the clinical presentation the provisional diagnosis of BCC and MM was made. An incisional biopsy was taken from the hyperpigmented patch and sent for histopathological examination and a fine needle aspiration cytology (FNAC) was performed for the nodular lesion.

**FIGURE 2:**
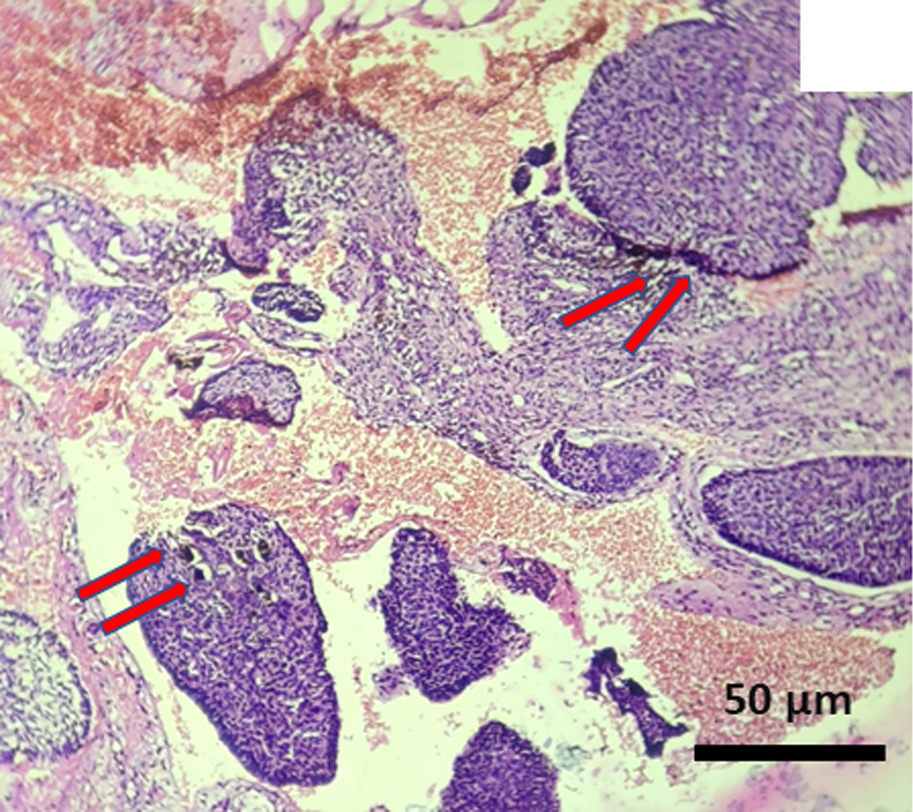
Photomicrograph showing nests and islands of basaloid epithelial cells with focal melanin pigmentation (represented by red arrows).

**FIGURE 3:**
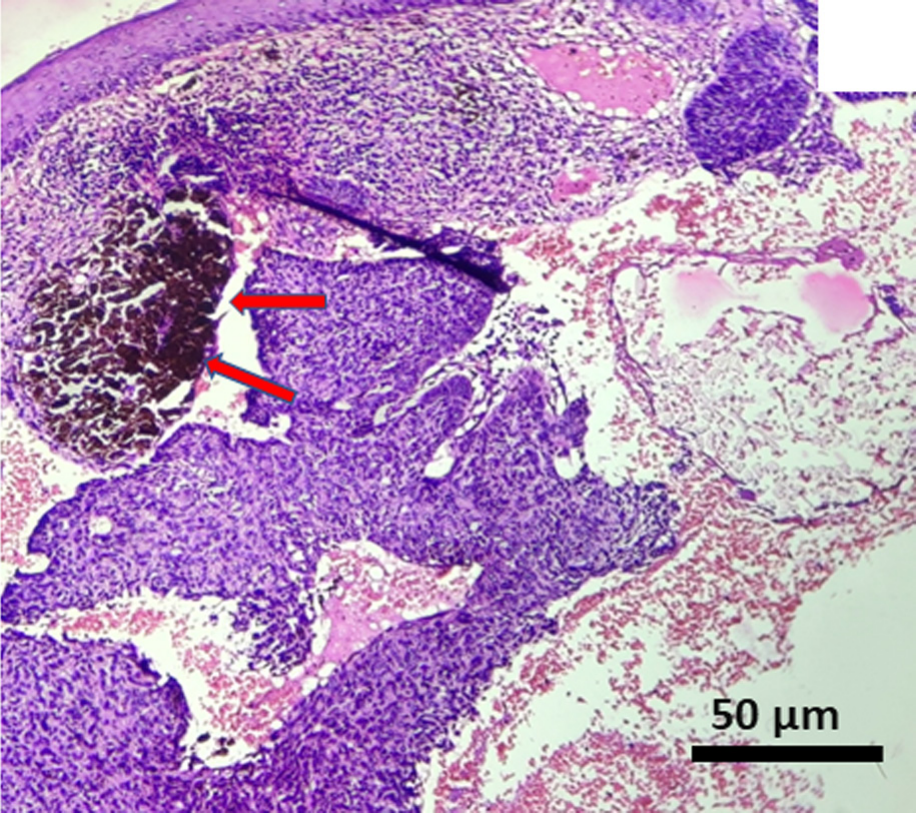
Photomicrograph showing extensive melanin pigmentation is noted within the tumor island (red arrow).

**FIGURE 4:**
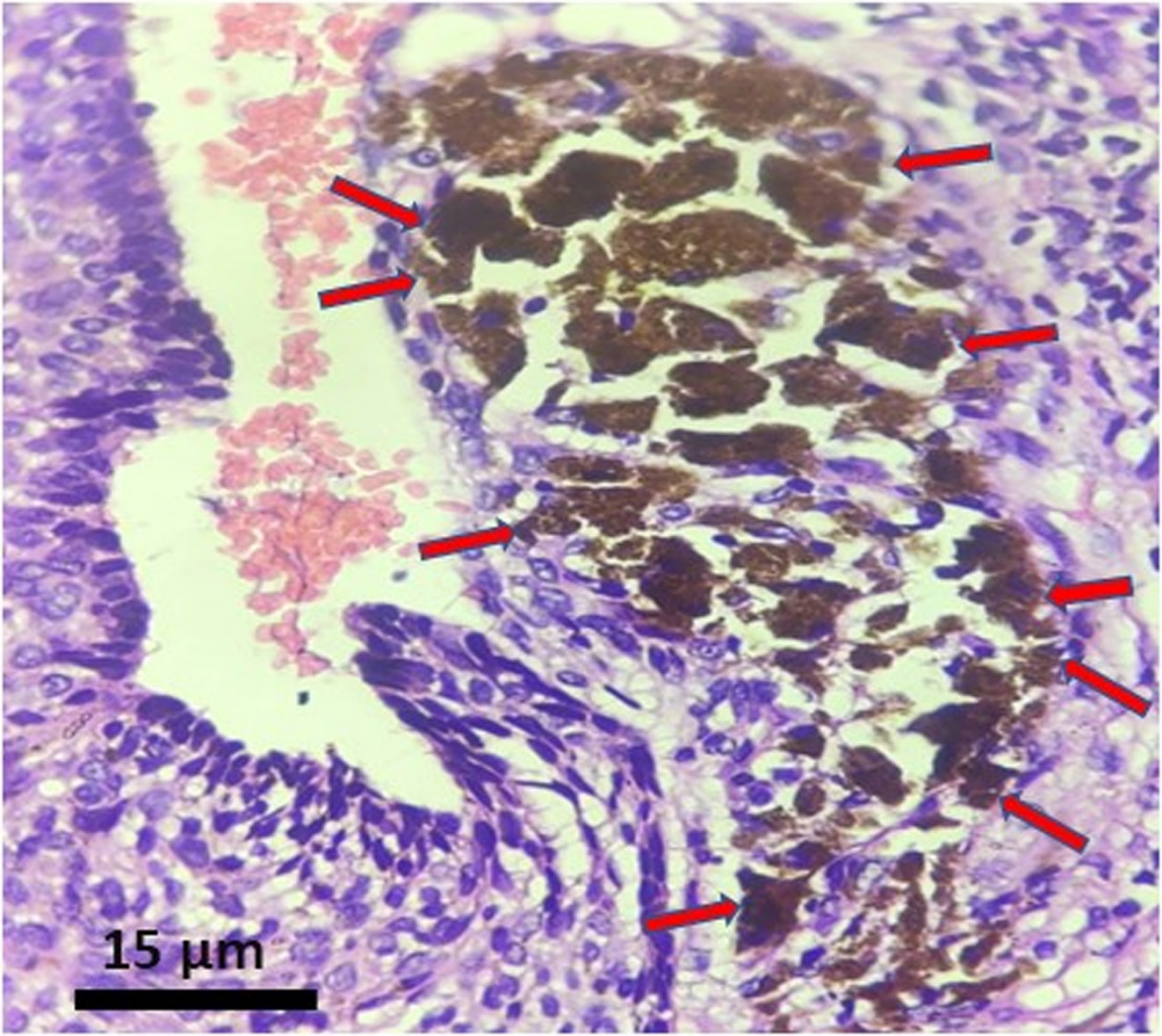
A high power view of melanin pigmentation amidst the tumor islands.

**FIGURE 5:**
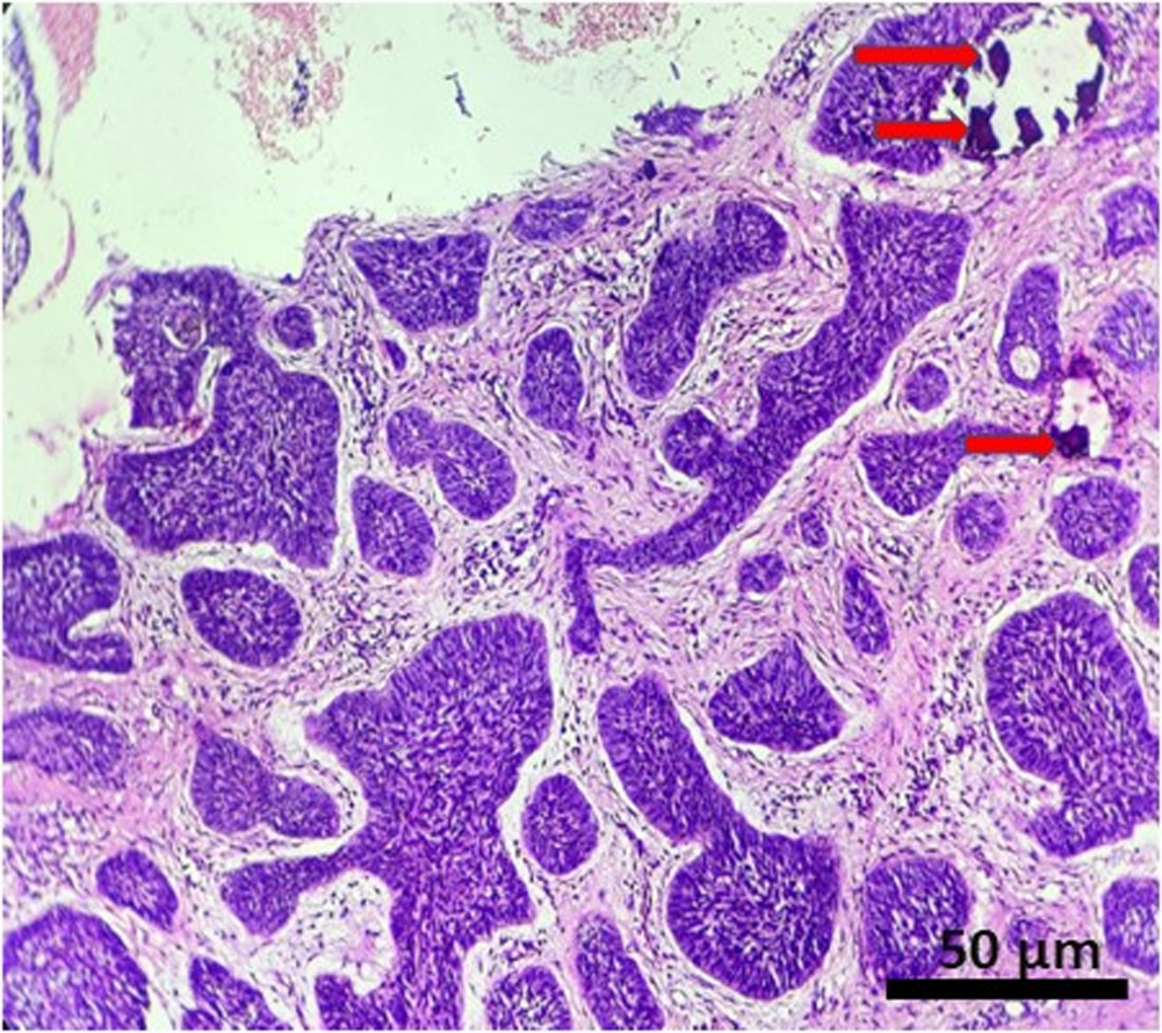
Photomicrograph showing lesional stroma with numerous islands, strands, and cords of basaloid epithelial cells with hyperchromatic nuclei and scanty cytoplasm. Focal areas of melanin pigmentation (red arrow) is seen.

The results of FNAC were nonconclusive. Whereas, the results of histopathological examination revealed lesional stroma, composed of characteristic basaloid cells with hyperchromatic nuclei which are arranged in nests, small cords, islands, and retraction cleft formation. (Figures 2, 3 and 5) Focal areas of melanin pigmentation within and around the tumor islands were also noted, which are represented by red arrows. (Figure 3). A high‐power view of melanin pigmentation amidst the tumor islands is depicted in Figure 4. The histopathological findings were consistent with the diagnosis of pigmented BCC.

The patient neither reported back to the hospital to collect the biopsy report nor we could communicate with the patient, so the present state of the lesion remains unknown.

## 3 DISCUSSION

Basal cell neoplasms are frequently seen in the head and neck region.^8^ Working classification of basal cell neoplasms has been proposed by Bajpai et al.^8^ in 2017, where BCC comes under basal cell neoplasms of oral epithelium.^8^ Other basal cell neoplasm includes tumors of odontogenic epithelium and salivary glands.^8^ Pigmented BCC is a rare variant, comprising up to 6% of the reported cases of BCC.^2^, ^9^ Ultraviolet radiation is the prime culprit for the development of BCC, although It occurs as a result of the interplay of various genetic and environmental factors.^1^, ^2^ The ultraviolet light brings about the mutation in various tumor suppressor genes like p53 and PTCH, thus causing BCC.^2^, ^7^ Exposure to ionizing radiations, arsenic, and various aromatic hydrocarbons can trigger mutation in growth regulatory genes and result in BCC.^9^ BCC can also be a part of syndromes like naevoid BCC syndrome (NBCCS), Bazex syndrome, and Rombo syndrome.^10^

The common age of presentation of BCC is in the 4th to 5th decade of life commonly affecting males with a male: female ratio of 3:2. The common predilected site for BCC is the middle one‐third of the face. There are reported cases of PBCC in the subareolar region of the nipples and the subungual areas.^11^ Bart RS et al. stated that the occurrence of PBCC is more common in dark‐eyed individuals when compared to blue‐eyed individuals.^12^Although metastasis is rarely seen in PBCC, Jung HJ et al. stated that higher chances of metastasis are noted in the PBCC which occurs in the nipple‐areolar region.^13^

Microscopically, BCC presents as nests, cords, and islands of basaloid cells with hyperchromatic nuclei with little or no cytoplasm. The cleft formation is frequently seen in the lesional stroma. In PBCC diffuse melanin pigmentation and melanophages are noted within the tumor islands and in the surrounding stroma.^2^, ^11^ Apart from histopathological evaluation, cytological examination, immunohistochemistry (IHC), and dermatoscopy are the other adjuncts in diagnosing pigmented PBCC^6^ IHC staining of PBCC shows positivity for Bcl 2, CD 10, Ber Ep‐4^14^, ^15^ whereas, tumors cells do not respond to HMB 45 which helps to differentiate PBCC from MM.^14^ S‐100 shows positivity to melanosomes and melanophages within the tumor islands, whereas the rest of the tumor cells do not respond to S‐100.^14^

The most common differential diagnosis for PBCC are melanocytic nevi, melanoma, and seborrheic keratosis. The less common ones are pilomatricoma basaloid squamous cell carcinoma and neuroendocrine tumors.^2^, ^11^, ^14^ The detailed description of differential diagnosis with differentiating histopathological features is mentioned in Table 1.

**TABLE 1:** Differential diagnosis of PBCC with differentiating features.^2^ ^,^ ^6^ ^,^ ^9^ ^,^ ^17^

Differential diagnosis	Differentiating histopathologic features
Naveus	The lesion shows nests/clusters of rounded/ovoid/epithelioid melanocytes with vesicular nuclei and pale cytoplasm and the presence of melanin pigment is noted in their cytoplasm
Melanoma	An isolated population of less cohesive melanocytes is noted throughout the lesional tissue. Cellular and nuclear pleomorphism is evident among the melanocytes. Few atypical mitoses also could be present
Seborrheic keratosis	The lesional tissue is composed of basaloid and squamous cells, keratinization is evident. The classic palisading appearance of basal cells is not evident
Basaloid squamous cell carcinoma	The lesion shows a higher degree of cellular and nuclear pleomorphism, enlarged nucleoli, atypical and abnormal mitoses, and a few of the cases show comedonecrosis which is not evident in PBCC
Pilomatricoma	The basaloid component of pilomatricoma may resemble BCC, but the characteristic ‘ghost cell’, multinucleated giant cells, and dystrophic calcifications makes it easily distinguishable from PBCC
Neuroendocrine tumor	It has a population of small blue round cells, which are more dispersed and nuclei covering the majority of the cell with little or no cytoplasm. Rosette formation is evident. These features help in distinguishing it from PBCC

The most accepted management for PBCC is surgical excision. Treatment with Mohs Micrognathic surgery (MMS) has shown promising results for the larger, aggressive deep‐seated lesions.^11^ Photodynamic therapy, intralesional injection of interferon‐alpha, and laser surgery are the newer alternative treatment options for PBCC.^11^

Maloney ME et al. (1992) ^16^ conducted a study to identify the subtypes of BCC. Out of 1039 cases of BCC that they evaluated 70 turned out to be PBCC. Among the 70 cases of PBCC, the most common form of presentation was the nodular/micronodular pattern (12.4%), followed by superficial pattern (7.2%).^16^

Many authors reported the presence of BCC in and around the eyes. Hornblass A et al. (1981) ^17^ reported nine cases of PBCC in the eyelid with a mean age of 67.4 years.^17^ Lin LK et al. (2008) ^18^ in the review reported 69 cases of BCC in the eyelid among Hispanics and out of them four cases were PBCC.^18^ The mean age of patients with PBCC was 63 years, and the lesion duration ranged from 7 months to 6 years.^19^

BCC is a skin cancer and is generally reported in a dermatological setup. The present case of PBCC was reported on a 76‐year‐old female with a chief complaint of toothache. The presentation of PBCC was the finding that the authors noticed while examining the patient. The patient restrained from any form of treatment and never reported back to the hospital. Therefore, any further progress on this case could not be mentioned. In the present case, we could not perform immunohistochemistry because there is no facility for immunohistochemistry in our country. We accept this as a limitation in our manuscript. Being a rare variant only a few cases of PBCC has been reported in the literature. The present case is being reported in a non‐dermatological setup. The details of the accessible reported cases of PBCC have been mentioned in Table 2.

**TABLE 2:** Detailed description of the accessible reported cases of PBCC in the literature.

Sr. No.	Author/Year	Age/Sex	Location	Presentation	Treatment	Follow‐up
1.	Casari A et al./(2011)^7^	Case 1 58/Male	Case 1 Temple (left side)	Case 1 Pigmented nodule, sharply demarcated, measuring about 6 mm	For all the cases Excisional biopsy	Follow‐up data not mentioned
		Case 2 73/Male	Case 2 Back of the patient	Case 2 A nodular lesion, irregular in shape, measuring about 8 mm with irregular borders		
		Case 3 44/ Male	Case 3 Forearm (right)	Case 3 A nodular pigmented lesion with sharply defined borders measuring about 2–3 cm		
		Case 4 60/Male	Case 4 Back of the patient	Case 4 A pigmented nodular lesion with central ulceration, irregular in shape, measuring about 1 cm		
2.	Jain M et al./(2012)^14^	55/Female	Dorsal aspect of left thigh	Non‐tender, focally ulcerated hyperpigmented polypoid growth developed over a period of 5 years. Size‐ (3 × 2 × 2) cm	Surgical excision.	Follow‐up period‐ 6 months. No recurrence
3	Coleman CI et al./(2013)^19^	Case 1 75 /Female	Case 1 Back of the patient	Case 1 Pink‐brown papule	Excisional biopsy (For both the cases)	Follow‐up data not mentioned
		Case 2 77/male	Case 2 Shoulder of the patient	Case 2 Brown and pink papule (Size not mentioned for both the cases)		
4	Deepadarshan K et al./(2013)^2^	Case 1 50/Male	Case 1 Right forehead	Case 1 Single pigmented papular lesion, depressed at the centre, with raised borders measuring about (5 × 6) cm	Case 1 Patient was referred to the oncologist for further management	Case 1 Follow‐up data not mentioned
		Case 2 68/Female	Case 2 Near the inner canthus of eye	Case 2 A solid, elevated pigmented lesion with raised borders measuring about (4 × 3) cm	Case 2 Surgical excision	Case 2 Healing uneventful. Follow‐up time not mentioned
5	Moraskar A/(2016)^11^	60/Female	Left side of the nose	Black elevated lesion, gradually developed over a period of 1 year, with well‐defined borders, measuring about (1.5 × 1.5) cm	Excisional biopsy with 2 mm surgical margin	Post excision, patient developed paresthesia at the operated site, which eventually got over in 6 weeks
6	Elfaituri SS/(2016)^10^	Male patient, age not mentioned	Scalp.	The initial lesion was presented 10 years back and was successfully excised. Multiple recurrent lesions, in the form of small plaques/nodules noted at the same site	Excisional biopsy	Follow‐up data of the present lesion (which is a recurrent one) not mentioned
7	Singh K et al./(2016)^20^	64/Male	Chest	An irregular hyperpigmented lesion which gradually increased in 3–4 months, measuring about (1.8 × 1) cm	Treatment details not mentioned	Follow‐up data not mentioned
8	Hasbún Acuña P et. al/(2016)^21^	55/Female	Lower lumbar region	Brown colored plaque with irregular borders, hard in consistency measuring about 5–7 cm in diameter	Surgical Excision	Follow‐up period‐ 1 year. No recurrence was noted
9	Abudu B et al./(2019)^22^	Case 1 74/Hispanic female	Case 1 Tip of the nose	Case 1 A persistent nodular blackish lesion with ulceration since 1 year. Size not specified	Case 1 Mohs surgery with forehead flap to treat the surgical wound	Follow‐up period for all three cases ‐ 3 months. No recurrence was noted
		Case 2 63/Hispanic male/	Case 2 Left nasal bridge	Case 2 Ulcerated plaque with red, fleshy areas present since 9 months. Size not specified.	Case 2 Mohs surgery with a full‐thickness graft to treat the surgical wound.	
		Case 3 77/Hispanic female	Case 3 Left breast	Case 3 Black nodule, persisting since 1 year with macular pigmentation and occasional bleeding measuring about (2 × 1) cm	Case 3 Mohs surgery with layered closure to treat the surgical defect	
10	Mudhar HS et al./(2020)^23^	60/Male	Left conjunctiva	A dark brown lesion, gradually developed over a period of 1 year, measuring about (3 × 3 × 2) mm	Local excision, with a clear margin of 4 mm	Follow‐up period‐ 2 months. No recurrence was noted
11	Present case	76/Female	Medial aspect of face	Irregular hyperpigmented patch, which gradually developed over a period of 3 years, measuring about (2.5 × 1.5) cm	The patient denied receiving any kind of treatment and never reported back to the hospital	No follow‐up data

## 4 CONCLUSION

BCC is one of the most commonly occurring nonmelanoma cancer, which presents in multiple forms. PBCC is more of the rare deceptive variant of PBCC and its diagnosis becomes challenging for the clinician. An under or over‐diagnosis of PBCC can affect the treatment outcome and the quality of life of the patient as well. The take‐home message to all clinicians including the dentists from this case report is any kind of deceptive presentation of lesion of the skin and face should be referred to a center where biopsy is available. A histopathological examination is mandatory to get a confirmatory diagnosis, before deciding on the treatment protocol.

## AUTHOR CONTRIBUTIONS

**Snehashish Ghosh:** Conceptualization; investigation; supervision; validation; writing – original draft; writing – review and editing. **Safal Dhungel:** Conceptualization; supervision; writing – review and editing. **Indu Bharkavi:** Conceptualization; writing – review and editing. **Bhawana Subedi Sapkota:** Data curation; investigation; supervision; writing – review and editing. **Prabesh Banstola:** Investigation; supervision; writing – review and editing.

## FUNDING INFORMATION

None.

## CONFLICT OF INTEREST STATEMENT

None.

## ETHICS STATEMENT

Ethical approval was not required from the institution, in accordance with our country's law, as this was a case report.

## CONSENT STATEMENT

Written informed consent was obtained from the patient to publish this case report in accordance with the journal's patient consent policy.
